# IL-1β-stimulated β-catenin up-regulation promotes angiogenesis in human lung-derived mesenchymal stromal cells through a NF-κB-dependent microRNA-433 induction

**DOI:** 10.18632/oncotarget.10683

**Published:** 2016-07-18

**Authors:** Jia Sun, Jintao Chen, Juan Cao, Tianxiang Li, Shaoxia Zhuang, Xiufeng Jiang

**Affiliations:** ^1^ Wuxi People's Hospital Affiliated to Nanjing Medical University, Wuxi 214023, Jiangsu, China

**Keywords:** lung injury, mesenchymal stem cell, angiogenesis, microRNA, Wnt/β-catenin

## Abstract

Considerable attentions have been focused on the treatment of lung injury using mesenchymal stem cells that can replenish damaged tissues including the blood vessels. In human lung-derived mesenchymal stem cells (hL-MSC), we investigated the potential role of an IL-1β-stimulated miR-433 pathway in angiogenesis *in vitro*. The expressions of miR-433 and its target genes were examined in cells treated with IL-1β. The angiogenic activity of hL-MSC was studied by cell migration and tube formation assays in which miR-433 levels were manipulated. The reporter assay and chromatin immunoprecipitation (ChIP) were also performed to analyze the underlying regulations. We found that the expression of miR-433 was enhanced in hL-MSC by IL-1β in a NF-κB dependent manner via a NF-κB binding site at its promoter region. The effects of IL-1β on promoting angiogenic activities in hL-MSC can be mimicked by the overexpression of miR-433 and were blocked by anti-miR-433. Mechanistically, our data suggested that miR-433 directly targets the 3′-UTR of Dickkopf Wnt signaling pathway inhibitor 1 (DKK1) mRNA and decreases its expression. Consistently, the expression of β-catenin, the major mediator of canonical Wnt pathway that is capable of inducing endothelial differentiation and angiogenesis, was upregulated by IL-1β through miR-433. Thus, increasing miR-433 expression by IL-1β in mesenchymal stem cells could stimulate their capacity of vascular remodeling for efficient repair processes, which may be utilized as a therapeutic target in patients suffering from severe lung injury.

## INTRODUCTION

Clinical presentation of diseases involved in the airways and pulmonary system is often caused by the damages in the cells of the lung due to environmental insults, infection or cancer, etc. The difficulty in reconstructing the complex-structured and functional organ such as the lung has limited the efficacy of treatment, and various types of lung diseases are implicated as devastating causes of morbidity and mortality [1, 2]. Because of their multipotent properties, mesenchymal stem cells (MSC) could be used as an efficient therapeutic option for patients with respiratory diseases such as chronic obstructive pulmonary disease (COPD) and acute respiratory distress syndrome (ARDS) [3–6]. A number of studies have supported the efficacy of MSC administration in various rodent model of lung injury. For example, bone marrow-derived MSC implantation protected lung damages induced by bleomycin in mice [7, 8]. In a mouse model of acute pulmonary inflammation induced by lipopolysaccharide (LPS), lung injury can also be attenuated by the treatment with MSC [9, 10]. Moreover, although more regulations and targeted approaches are probably needed [11], the early results of some clinical trials favored the safety and benefit of MSC therapy specifically in some types of lung diseases such as COPD [4].

While the mechanisms of MSC actions have not been fully elucidated, MSC are capable of differentiating into epithelial or endothelial cells that are important for positive tissue remodeling in response to lung injury. In particular, neovascularization is an essential step for healing process and the direct contribution of MSC to newly formed vascular cells could result in improved pulmonary perfusion necessary for healing. This stimulation of angiogenesis in MSC could be mediated, at least in part, by a canonical Wnt signaling through β-catenin. It has been reported that β-catenin is indispensable for vasculogenic differentiation of human dental pulp stem cells [12]. Exosomes from human umbilical cord M can enhance angiogenesis through a β-catenin-dependent pathway [13]. In LPS-induced acute lung injury model, overexpression of β-catenin in MSC provided further improvements in alveolar epithelium regeneration and pulmonary permeability [14]. Additionally, MSC can exert their beneficial effects through orchestrating optimal microenvironment for organ repair. Accumulated data have suggested MSC possess immunomodulatory functions [15–17] which may contribute to their therapeutic potential for inflammation-driven lung diseases. In this context, despite of their immune-privileged status, MSC could still be influenced by inflammatory cytokines through a variety of signaling pathways, which can promote important functions of MSC including angiogenesis [18–20]. The capacity of cytokine-stimulated angiogenesis in MSC could thus serve to facilitate lung repair, and the better characterization of the underlying mechanisms may provide novel insights for the refinement of MSC therapy.

Regarding the possible downstream signaling of cytokine-stimulated MSC, the implication of a major class of molecular modulators, such as microRNAs (miR), has not been previously well-explored. As post-transcriptional regulators, microRNAs are expressed from non-coding genome regions and repress the stability and/or translation of target genes by specifically binding on the 3′ untranslated regions (UTR) of their mRNAs [21, 22]. The important roles of microRNAs have been implicated in both angiogenesis and mesenchymal stem cell [23–25]. In the current study, we examined human lung-derived mesenchymal stem cell (hL-MSC) stimulated by inflammatory cytokine IL-1β. We found miR-433 was specifically upregulated, which in turn led to increased β-catenin level through the inhibition of Dickkopf Wnt signaling pathway inhibitor 1 (DKK1) expression in hL-MSC. Finally, the enhanced miR-433 expression was required for IL-1β-induced angiogenesis of hL-MSC, highlighting miR-433 as a tractable target for therapeutic applications in improving lung repair by mesenchymal stem cells.

## RESULTS

### IL-1β-stimulated miR-433 decreases DKK1 expression in hL-MSC

**Figure 1:**
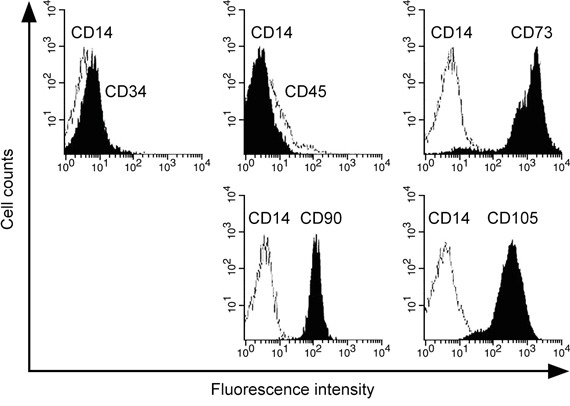
Identification of human lung-derived MSC Cells were characterized by flow cytometry using FITC- or PE-conjugated antibodies against negative surface markers CD14, CD34, CD45 and positive surface markers CD73, CD 90, CD105.

**Figure 2:**
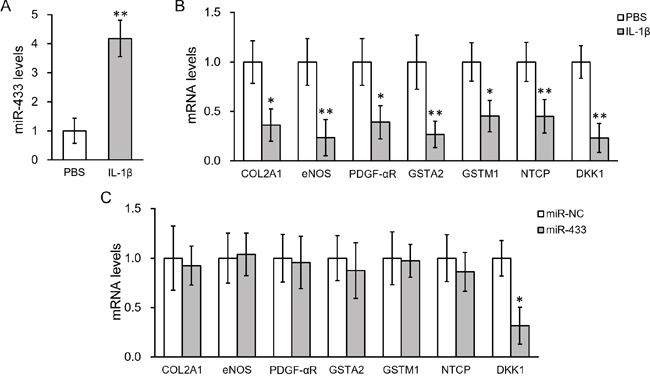
IL-1β treatment upregulated miR-433 and down-regulated DKK1 expressions in hL-MSC **A.** Levels of miR-433 in hL-MSC after IL-1β treatment, with PBS as control. **B.** mRNA levels of genes known to be inhibited by IL-1β after IL-1β treatment, with PBS as control. **C.** mRNA levels of genes in hL-MSC transfected with either miR-negative control (NC) or miR-433. Values were mean ± SD from three independent experiments. ** P < 0.01, * P < 0.05 vs PBS or miR-NC, respectively.

The method to obtain MSC from bronchoalveolar lavage (BAL) of human patients has been previously shown [26, 27]. We therefore isolated and confirmed the progenitor cell identity of human lung-derived MSC, which was shown negative for CD14, CD34 and CD45 whereas positive for CD73, CD90 and CD105 (Figure 1). We then analyzed the expression of miR-433 in the cultured MSC treated with 10 ng/ml IL-1β for 24 hours. MiR-433 expression was found highly stimulated by IL-1β compared with PBS control-treated hL-MSC (up to 4 fold compared to PBS control, Figure 2A), suggesting a potential function of miR-433 in response to IL-1β in hL-MSC. To assess the possible target genes that could be suppressed by miR-433 in hL-MSC, we investigated the expression of genes that are known to be inhibited by IL-1β, such as collagen type 2 (COL2A1), endothelial nitric oxide synthase (eNOS), PDGF-alpha receptor subunit (PDGF-αR), glutathione S-transferase GSTA2 and GSTM1, and sodium-taurocholate cotransporting polypeptide (NTCP) [28–32]. Consistent with prior data, these genes were all down-regulated by IL-1β (Figure 2B). However, an overexpression of miR-433 in MSC didn't have any effect as IL-1β stimulation (Figure 2C). In contrast, Dickkopf Wnt signaling pathway inhibitor 1 (DKK1), a negative regulator of Wnt/β-catenin signaling, was inhibited in both conditions (Figures 2B and 2C), suggesting that miR-433 upregulation might be a novel mediator of IL-1β signaling in hL-MSC potentially via a modulation of Wnt pathway.

### IL-1β promotes angiogenic activity of hL-MSC through miR-433 upregulation

**Figure 3:**
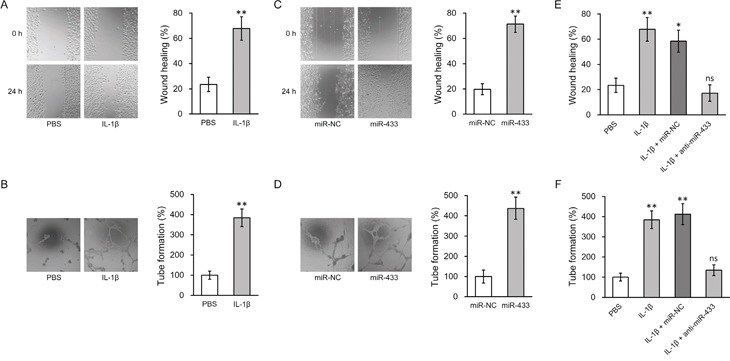
miR-433 was required for IL-1β-induced enhancement of angiogenesis in hL-MSC derived endothelial cells **A.** and **B.** Wound healing (A) and tube formation (B) assays were performed in hL-MSC derived endothelial cells treated with PBS or IL-1β. **C.** and **D.** Wound healing (C) and tube formation (D) assays were performed in hL-MSC derived endothelial cells transfected with miR-NC or miR-433. **E.** and **F.** hL-MSC derived endothelial cells treated with PBS or IL-1β were also transfected with either miR-NC or anti-miR-433, followed by wound healing (E) and tube formation (F) assays to assess their angiogenic capacity. Values were mean ± SD from three independent experiments. ** P < 0.01, * P < 0.05, ns not significant vs respective control.

It is known that canonical Wnt signaling through β-catenin plays essential roles for both differentiation and function of the endothelial cells. To assess the ability of MSC to differentiate into endothelial cell, we performed FACS analysis based on the cell surface marker CD31. Upon incubation with 20 ng/ml bFGF, cultured hL-MSC were differentiated into endothelial cells as previously reported [33], which could be further enhanced by either IL-1β or miR-433 overexpression (Figure S1). As angiogenesis is an important endothelial function for lung tissue regeneration, we further assessed IL-1β-treated MSC for the ability of cell migration and tube formation, two *in vitro* assays for the evaluation of angiogenesis. We first created a scratch in the endothelial cell monolayer, and then monitored the wound closure by migrating cells in the absence or presence of 10 ng/ml IL-1β. An approximate two fold increase in cell migration by IL-1β treatment was observed in hL-MSC-derived endothelial cells (Figure 3A). We next mixed cells within three dimension cultures to induce capillary-like structures. Incubation with IL-1β resulted in a dramatic increase in tube formation (Figure 3B). Likewise, miR-433 overexpression enhanced the angiogenic potential of hL-MSC-derived endothelial cell culture, shown by the increased wound healing and tube forming activities (Figure 3C-3D), which implied that increasing miR-433 expression may be involved in IL-1β-activated cell functions in hL-MSC. To directly test this hypothesis, we examined miR-433 dependency for IL-1β-stimulated angiogenesis by anti-miR-433. Compared to control miR oligos, hL-MSC-derived endothelial cells transfected with anti-miR-433 failed to response to IL-1β, with regard to both wound healing and tube formation capabilities. Transfection of scrambled control miR in hL-MSC-derived endothelial cells didn't affect the ability of cell migration induced by IL-1β. However, anti-miR-433 transfection abolished the increase in wound healing (Figure 3E). Furthermore, tube formation in IL-1β-treated cells was reversed only in the presence of anti-miR-433 (Figure 3F). Altogether, these data suggested that the stimulating effects of IL-1β on the angiogenic activity of hL-MSC-derived endothelial cells are mediated through miR-433.

### IL-1β-stimulated miR-433 represses DKK1 expression through 3′UTR

**Figure 4:**
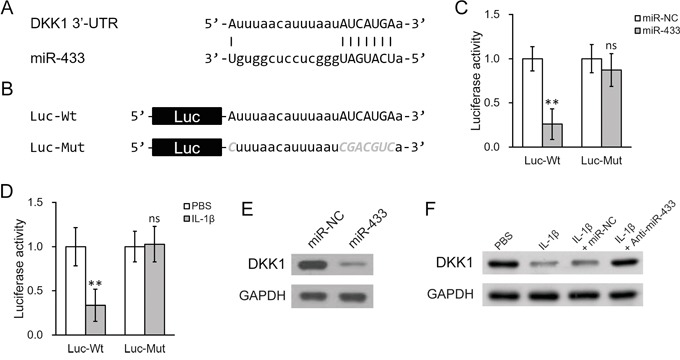
IL-1β treatment upregulated miR-433, which directly targeted the 3′-UTR on DKK1 mRNA in hL-MSC **A.** Sequence of the putative miR-433 targeting site (capitalized) on the 3′-UTR of DKK1 mRNA. **B.** Wild type (-Wt) or mutated (-Mut) versions of putative targeting sequence from the 3′-UTR of DKK1 mRNA were fused after the downstream of a luciferase reporter (Luc) open reading frame. **C.** and **D.** Luciferase activities of Luc-Wt and Luc-Mut constructs were measured in hL-MSC after transfection with either miR-NC or miR-433 (C), or treatment with either PBS or IL-1β (D). **E.** DKK1 protein levels in hL-MSC after transfection with either miR-NC or miR-433. **F.** hL-MSC treated with PBS or IL-1β were also transfected with either miR-NC or miR-433 inhibitor (anti-miR-433), followed by Western blot analysis to examine DKK1 protein levels. Values were mean ± SD from three independent experiments. ** P < 0.01, * P < 0.05, ns not significant vs PBS or miR-NC, respectively.

It is commonly observed that a complementary sequence of microRNAs resides at the 3′ untranslated region (UTR) of the target gene. We therefore analyzed the 3′-UTR of DKK1 mRNA. It was found that a potential binding pair may exist between miR-433 and 3′-UTR of DKK1 mRNA based on computational analysis (Figure 4A). Luciferase reporter assay was then carried out to test the specific binding by examining the activity of luciferase under the control of 3′-UTR of DKK1 (Figure 4B). As shown in Figure 4C, co-transfection of miR-433 greatly diminished the luciferase activity of the reporter containing wild type sequence of 3′-UTR of DKK1 mRNA. However, this decrease was not seen when the predicted binding site for miR-433 was mutated. Similar modulation was found in cells treated with IL-1β. IL-1β decreased the luciferase activity of wild type but not the mutant 3′-UTR of DKK1 (Figure 4D). We then performed Western blotting to confirm if the results in the reporter study correspond to the changes of endogenous DKK1 protein levels. First, transfection of miR-433 in hL-MSC led to a decrease of DKK1 protein (Figure 4E). Second, IL-1β lowered DKK1 protein as well (Figure 4F). Finally, the repressed DKK1 protein by IL-1β could be specifically rescued by a blocking oligonucleotide for miR-433 (Figure 4F, anti-miR-433). Taken together, these data demonstrated that IL-1β-stimulated miR-433 could decrease DKK1 mRNA and protein levels in hL-MSC, possibly through a direct binding to the 3′-UTR region of DKK1 mRNA.

### IL-1β-induced miR-433 expression depends on NF-кB activation

**Figure 5:**
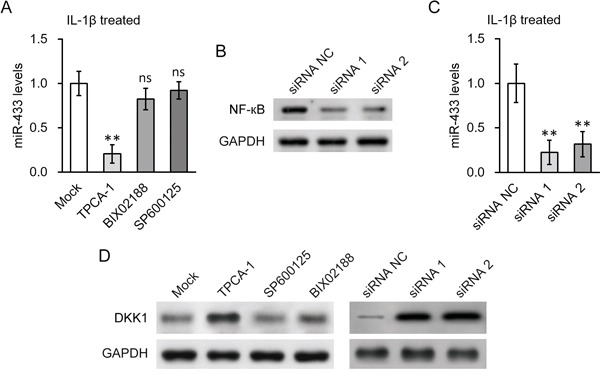
NF-кB activation was required for IL-1β induced upregulation of miR-433 in hL-MSC **A.** Levels of miR-433 in the presence of IL-1β were examined in hL-MSC after treatment with either NF-кB inhibitor TPCA-1, p38 inhibitor BIX02188 or JNK inhibitor SP600125, respectively. **B.** and **C.** Levels of NF-кB protein (B) and miR-433 (C) in hL-MSC following NF-кB siRNA knock-down were examined by Western blot and RT-PCR, respectively. **D.** Protein levels of DKK1 in hL-MSC in the presence of IL-1β were examined by Western blot, following treatment with the inhibitors used in (A) or NF-кB siRNA knock-down used in (B). Values were mean ± SD from three independent experiments. ** P < 0.01, ns not significant vs mock or siRNA NC, respectively.

We next investigated the molecular mechanisms underlying the induction of miR-433 by IL-1β. Given the strong association of IKK/NF-κB pathway with inflammation signaling, we hypothesized that NF-κB activation is required for the stimulation of miR-433 expression by IL-1β. In agreement with this thought, an inhibitor of IKK, TPCA-1, significantly blocked the miR-433 induction by IL-1β in hL-MSC (Figure 5A). As controls, inhibitors to p38MAP kinase (BIX02188) or JNK (SP600125) pathways had no effect. The result was further supported by genetic approaches using siRNAs knockdown of NF-κB (Figure 5B). The induction of miR-433 expression was attenuated when NF-κB was knocked down by two different siRNA oligos (Figure 5C). Consistent with our previous findings, miR-433-dependent DKK1 repression in hL-MSC in the presence of IL-1β was also prevented by either IKK inhibition or NF-κB knockdown (Figure 5D).

**Figure 6:**
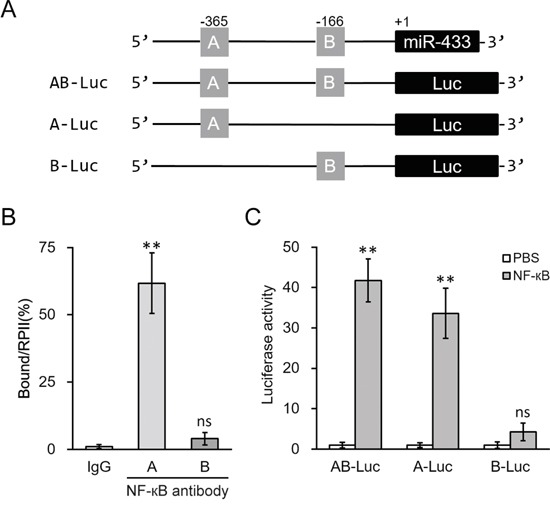
NF-κB induced miR-433 expression by directly binding to its promoter region **A.** Promoter region of human miR-433 contains two putative binding sites for NF-κB, which was then clone to the upstream of a luciferase reporter (Luc) open reading frame. **B.** Binding of NF-κB to the promoter of miR-433 in hL-MSC was examined by ChIP assay using control IgG or NF-κB antibody. **C.** Luciferase activities of AB-Luc, A-Luc and B-Luc constructs were measured in hL-MSC after treatment with either PBS or NF-κB. Values were mean ± SD from three independent experiments. ** P < 0.01, ns not significant vs control IgG or PBS, respectively.

To further validate direct binding of NF-κB to the promoter region of miR-433, we examined the upstream region of human miR-433 gene (miRBase Accession MI0001723). The promoter region sequence was retrieved from GeneBank (NC_000014.9[100881121..100881885]). Two potential NF-κB responsive DNA-binding sites, bearing the consensus sequences-GGRNNYYC (R purine; Y pyrimidine; N any base), were found within the promoter region and named as region A (-365) and B (-166), respectively (Figure 6A). In ChIP assays performed in hL-MSC, NF-κB specifically bound to region A, whereas minimal binding of NF-κB was seen at region B (Figure 6B). Consistently, the transcriptional activity of the miR-433 promoter reporter in hL-MSC was stimulated by NF-κB, but only in the presence of intact region A, as determined by luciferase reporter assay shown in Figure 6C. Thus, NF-κB activation appeared to be essential for the miR-433 induction following IL-1β treatment in hL-MSC, through binding specific elements of miR-433 promoter.

### IL-1β-stimulated miR-433 enhances β-catenin expression in hL-MSC

**Figure 7:**
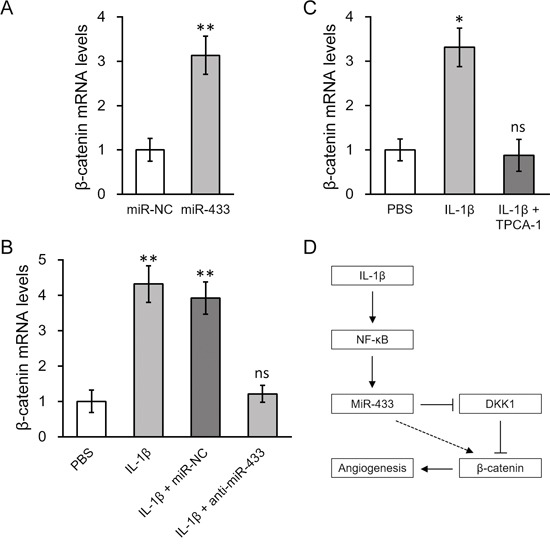
β-catenin expression was upregulated by IL-1β induced miR-433, in a NF-κB dependent manner **A.** Levels of β-catenin mRNA in hL-MSC transfected with either miR-NC or miR-433. **B.** hL-MSC treated with PBS or IL-1β were also transfected with either miR-NC or anti-miR-433, followed by RT-PCR analysis to examine β-catenin mRNA levels. **C.** Levels of β-catenin mRNA in hL-MSC treated with PBS, IL-1β or IL-1β + NF-кB inhibitor TPCA-1. Values were mean ± SD from three independent experiments. ** P < 0.01, * P < 0.05, ns not significant vs miR-NC or PBS, respectively. **D.** A schematic diagram illustrating the mechanism of IL-1β-stimulated β-catenin up-regulation, mediated by NF-κb-dependent miRNA-433 induction, to promote angiogenesis in hL-MSC.

DKK1 is known to antagonize Wnt/β-catenin signaling [34, 35], therefore repression of DKK1 by IL-1β through miR-433 may activate Wnt/β-catenin pathway. To test this hypothesis, we transfected miR-433 into hL-MSC, and found that it not only significantly increased β-catenin mRNA expression up to 3 fold compared to control miR oligos (Figure 7A), but also greatly enhanced nuclear import of β-catenin (Figure S2). IL-1β treatment triggered an even higher response in the level of β-catenin, which could be then completely abolished by anti-miR-433 (Figure 7B). Consistent with blocking miR-433 simulation by TPCA-1, the inhibition of NF-κB signaling by TPCA-1 also attenuated the increased expression level of β-catenin in hL-MSC following treatment with IL-1β (Figure 7C). A summary of our major findings are presented as follows: in hL-MSC IL-1β stimulates NF-κB-dependent miR-433 expression, which in turn induces β-catenin activation in promoting angiogenesis through the repression of DKK1 (as depicted in Figure 7D).

## DISCUSSION

The main repair function of stem/progenitor cells includes the ability of multipotent differentiation capacity to replenish the damaged tissues. Particular in treating lung diseases, the reestablishment of functional microvasculature to the injured lung is a key step for efficient repair, which could be facilitated by MSC. Recently, it has been suggested that MSC can directly or indirectly promote angiogenesis, in which Wnt/β-catenin pathway may play an essential role. It was shown that direct activation of Wnt/β-catenin in postnatal mesenchymal stem cells can sufficiently induce vessel formation both *in vitro* and *in vivo*. β-catenin deficiency completely abolished the ability of MSC to differentiate into vascular cells [12]. Interestingly, MSC-derived extracellular vesicles containing Wnt4 was able to enhance the migration and tube formation of endothelial cells through promoting β-catenin activity [13]. Such pro-angiogenic function of Wnt/β-catenin in MSC could be essential in repairing injured lung. In a prior study using an animal model of ARDS, the therapeutic effect of Wnt/β-catenin activation has been directly demonstrated. The overexpression of β-catenin in engrafted MSC greatly helped the regeneration of impaired lung tissue [14]. Thus, a strategy to increase β-catenin signaling in MSC may provide clinical benefit for treating lung diseases by specifically promoting the angiogenic potential of the stem cells.

We have demonstrated hereby that IL-1β-stimulated pathway could be an option to induce β-catenin-dependent angiogenesis of MSC. Through NF-κB activation, IL-1β increased miR-433 expression in hL-MSC. This effect was mainly dependent on the NF-κB-binding site at the promoter region of miR-433. In turn, the negative regulator of Wnt/β-catenin signaling, DKK1 was repressed by miR-433 targeting on the 3′-UTR of its mRNA, which then led to β-catenin upregulation. Finally, the importance of miR-433 has been implicated in angiogenic activity of hL-MSC. Overexpression of miR-433 enhanced, whereas anti-miR-433 blocked IL-1β-induced angiogenic effects in endothelial cell migration and tube-forming activity. These data collectively highlighted miR-433 as a potential molecular target for therapeutic manipulation of MSC in lung repair (Figure 7D).

The potential regulatory function of miR-433 in Wnt signaling has been previously suggested. Modulated by MEF2A in a large non-coding RNA cluster, miR-433 inhibited the expression of secreted Frizzled-related proteins (sFRPs) in skeletal muscle cells. Accordingly, the upregulation of miR-433 was found to decrease the inhibitor of Wnt signaling sFRP2, therefore activating β-catenin-dependent myogenic differentiation. [36] Consistent with this finding, our data supported that miR-433 expression positively correlated with β-catenin expression. Specifically, the link was connected through another antagonist of Wnt/β-catenin signaling DKK1, and we identified and confirmed a direct binding site of miR-433 on the 3′-UTR of DKK1 mRNA. These results suggested that miR-433 may exert its action on Wnt/β-catenin signaling through multiple targets.

Another novel finding of this study was probably the demonstration of an important role of miR-433 in promoting MSC functions following its differentiation. Namely, miR-433 appeared to be involved in IL-1β-stimulated angiogenesis of hL-MSC. MicroRNAs are known to participate in various biological processes in stem/progenitor cells including cellular differentiation. Notably, miR-433 modulation has been observed in several cases of lineage commitment in stem cells. A prior study has investigated osteoblast differentiation of MSC linage C3H10T1/2, in which miR-433 exhibited a suppressive role [37]. Additionally in embryonic striatal stem cells, insulin growth factor (IGF)-1-induced miR-433 was proposed as a fate switching player of striatal precursors towards proliferation and lineage differentiation [38]. On the other hand, there is very limited information regarding miR-433 in the blood vessel formation. Although a role of miR-433 in modulating endothelial redox homeostasis has been previously described [39], whether miR-433 could be a determining factor for endothelial differentiation is completely unknown. Studies focusing on endothelial-specific miR-433 expression in the development of vasculature are needed to address this question, and further study into the healing processes could be informative for the understanding of unique roles of miR-433 in stem cell biology.

Given the essential functions of microRNAs in various types of physiological processes, there is still lack of information available for the transcriptional modulation of microRNA expression. Our reporter assay and ChIP experiments found that IL-1β induced miR-433 expression through a conventional transactivation of NF-κB at the promoter of miR-433. Several classes of microRNAs contain the canonical NF-κB responsive element in their promoter regions [40–42], and our study have identified a similar binding of NF-κB p65 subunit to the promoter of miR-433 at -365 from the start site. Inhibition of NF-κB activity diminished miR-433 stimulation by IL-1β in hL-MSC. Interestingly, derived from the same gene cluster with miR-433 [43], miR-127 was found to be reduced by IL-1β in osteoarthritic human cartilage [44]. Thus, a co-regulation of paired miRNAs by the same transcription factor can lead into differential expressions, implementing a prior evolution theory about the clustered miRNA genes [43].

Whether miR-433 induction could lead to increased neovascularization and improved lung repair *in vivo* is still unclear. To test this hypothesis, the administration of miR-433-manipulated MSC to lung injury models would be important. These results may potentially differentiate between the multiple functions of MSC for treating lung injury and fibrosis, such as suppression of inflammation and production of reparative growth factors. In addition, our study of *in vitro* angiogenesis assays did not discount the possibility that a β-catenin-independent pathway also contributes to the angiogenic activation of hL-MSC by miR-433/IL-1β. Future studies are required to determine the dependency of miR-433 functions on Wnt/β-catenin signaling. By linking β-catenin and miR-433, both of which have been associated with tumor progression, our findings may also provide mechanistic insights for the link between inflammation and pathogenesis of cancer. Investigation of such issue in cancers with miR-433 elevation will be of particular interests to study if the potential increase of β-catenin activity would contribute to tumorigenesis in these cases.

## MATERIALS AND METHODS

### Isolation and identification of human lung-derived MSC

Mesenchymal stem cells were derived from cells isolated from bronchoalveolar lavage (BAL) of patients receiving lung transplant in Wuxi People's Hospital Affiliated to Nanjing Medical University following procedures as previously described [26, 27], and written informed consent forms were acquired from patients before the study. In brief, cells obtained from BAL fluid were filtered through cell strainer to remove particulate material and mucus. The cell pellets after washing were then maintained in DMEM culture media supplemented with penicillin/streptomycin and 10% fetal bovine serum at 37°C in 5% CO_2_ and used at passages 2-6. The characterization of surface markers as hL-MSC was performed by flow cytometry using FITC- or PE-conjugated antibodies against CD31, CD34, CD45, CD14, CD73, CD90, and CD105 (eBioscience, San Diego, CA, USA). The negative stained cells by isotype type control antibody, CD14 were used to optimize photo-multiplier tube and compensation in the analysis using BD-FACScan. The data were analyzed with Flowjo. This study was approved by the ethics committee of Wuxi People's Hospital Affiliated to Nanjing Medical University under the IRB number WXPH075311Z.

### MicroRNA transfection and measurement

The *mir*Vana miRNA mimic and antisense set for human miR-433 (MH10774) from Applied Biosystems (Carlsbad, CA, USA) were transfected into the cells based on manufacturer's instructions. The *mir*Vana miRNA Isolation Kit (AM1561, Applied Biosystems) was used to isolate total miRNA, and expression levels of miR-433 were then determined by pri-miRNA assay kit (Hs03303744_pri, Applied Biosystems) and mature miRNA assay kit (478102_mir, Applied Biosystems) according to manufacturer's instructions.

### mRNA extraction and quantitative PCR

Total mRNA was isolated from cultured cells at the indicated conditions using the RNeasy kit (Qiagen, Valencia, CA, USA) and was reverse transcribed to complementary cDNAs with Superscript II (Biorad, Hercules, CA, USA) according to manufacturer's instructions. Specific primers used for human gene transcripts are described as following: *IL-1β*, forward 5′-ATG ATG GCT TAT TAC AGT GGC AA-3′ and reverse 5′-GTC GGA GAT TCG TAG CTG GA-3′; *Col2A1*, forward 5′-TGG ACG ATC AGG CGA AAC C-3′ and reverse 5′-GCT GCG GAT GCT CTC AAT CT-3′; *NOS3*, forward 5′-TGA TGG CGA AGC GAG TGA AG-3′ and reverse 5′-ACT CAT CCA TAC ACA GGA CCC-3′; *PDGFRA*, forward 5′-TTG AAG GCA GGC ACA TTT ACA-3′ and reverse 5′-GCG ACA AGG TAT AAT GGC AGA AT-3′; *GSTA2*, forward 5′-TAC TCC AAT ATA CGG GGC AGA A-3′ and reverse 5′-TCC TCA GGT TGA CTA AAG GGC-3′; *GSTM1*, forward 5′-TCT GCC CTA CTT GAT TGA TGG G-3′ and reverse 5′-TCC ACA CGA ATC TTC TCC TCT-3′; *SLC10A1*, forward 5′-AAG GAC AAG GTG CCC TAT AAA GG-3′ and reverse 5′-TTG AGG ACG ATC CCT ATG GTG-3′; *DKK1*, forward 5′-ATA GCA CCT TGG ATG GGT ATT CC-3′ and reverse 5′-CTG ATG ACC GGA GAC AAA CAG-3′; *CTNNB1*, forward 5′-AGC TTC CAG ACA CGC TAT CAT-3′, reverse 5′-CGG TAC AAC GAG CTG TTT CTA C-3′ ; *GAPDH*, forward 5′-ACA ACT TTG GTA TCG TGG AAG G-3′, reverse 5′-AAG TGG TCG TTG AGG GCA ATG-3′. A SYBR Green dye-based detection method was performed using the SYBR Green PCR Master Mix assay (Applied Biosystems). A series of duplicate dilutions of cDNA from control samples were used to optimize the standard curve and validate the melting curves for each primer set. Triplicated PCR reactions were carried out for each sample. GAPDH was used as a housekeeping gene for normalization of the expression levels.

### Luciferase assay

The 3′-UTR region of DKK1 mRNA containing the putative miR-433 targeting site (wild type or mutant sequences) was fused after the open reading frame of pGL3 luciferase reporter plasmid (Luc). The promoter region of human miR-433 contains two potential binding sites for NF-κB, and has been cloned into pGL3 luciferase reporter plasmid at the upstream of Luc open reading frame. The constructs with individual binding site-deleted portions were also obtained. hL-MSC were transfected with the reporters in the absence or presence of miRNA oligos. The activity was then measured in the absence or presence of IL-1β stimulation with a Dual-Luciferase Assay System (Promega, Madison, WI, USA).

### Western blotting

Western blotting was performed in cultured cells following various treatments. The protein lysates were measured by BCA assay and the same amount of proteins was resolved on SDS-PAGE followed by an electric transfer to a PVDF membrane. The blots after blocking were incubated with primary antibodies, including DKK1 (Abcam, Cambridge, MA, USA), p65 NF-κB (Santa Cruz Biotechnology, Santa Cruz, CA, USA), and GAPDH (Santa Cruz Biotechnology). The blots were then incubated by HRP conjugated secondary antibody, and signals were developed by ECL-based imaging system.

### Chromatin immunoprecipitation (ChIP)

The binding of NF-κB to the promoter of miR-433 was examined by ChIP assay in hL-MSC. In brief, cells were cross-linked with 1% formaldehyde and the genomic DNA was then sheared into an average size of 400 bp followed by an immunoprecipitation by anti-NF-κB p65 (Santa Cruz Biotechnology, sc-109). An immunoprecipitation with nonspecific IgG was also used as negative control. Eluted DNA from the immunoprecipitation was analyzed by quantitative PCR. The ChIP-PCR primers were used to span each of two putative NF-κB binding sites in the promoter region of miR-433. The result of binding was calculated as the fold enrichment of the ChIP sample relative to the control IgG samples.

### Analysis of angiogenesis potential of hL-MSC

For determining angiogenic activity, hL-MSC were mixed with Matrigel (BD Bioscience, Franklin Lakes, NJ, USA) and seeded on culture wells supplemented with 20 ng/mL bFGF (R&D Systems, Minneapolis, MN, USA). The medium was replaced every day. The differentiation of MSC towards to endothelial lineage was observed as the cells formed branches and tube-like extensions similar as endothelial capillaries. To assess the ability of cell migration [45], MSC after the induction of endothelial differentiation were trypsinized, resuspended and plated in new culture wells until confluency. A scratch was then generated by a 200 μL pipette tip. The migrating cells into the scratches after 24 hours were monitored and the distance between the front edges of migrating cells were then compared to the cell boundary at time zero. To further investigate the neovascularization efficiency, we performed the *in vitro* tube formation assay as previously described [45]. Briefly, hL-MSC After the treatments were seeded onto the pre-solidified Matrigel at 2,000 cells per well. The tubes formed 20 hours later were imaged with a microscope, and quantified by the measurement of the tube length in five random areas in Image J from NIH. Experiments were repeated at least three times and the results were calculated as the relative fold to the control treated cells.

### Statistical analysis

All data in graphs are generated from at least three independent experiments, and expressed as the means ± SD as indicated. Prism was used to evaluate the data for statistical significance by two-tailed student t tests (Figure 2, 3, 6A, 7, and 8) and one-way ANOVA (Figure 4, 5, 6B-6C and 9). P < 0.05 was considered as statistically significant.

## CONCLUSIONS

There is a growing interest to facilitate the efficacy and safety of MSC in treating lung injury diseases. Based on our results, a Wnt/β-catenin signaling may be critical for the angiogenic potential of MSC associated with the regeneration processes. Specifically, the current findings suggest that a microRNA, miR-433, is involved in IL-1β-induced MSC angiogenesis by increasing β-catenin expression through DKK1 repression. Thus, the manipulation of miR-433 pathway in MSC could be useful for an efficient lung repair.

## SUPPLEMENTARY FIGURES


