# Supplementary material for: IL-1β-stimulated β-catenin up-regulation promotes angiogenesis in human lung-derived mesenchymal stromal cells through a NF-κB-dependent microRNA-433 induction
Source: Oncotarget. 2016 Jul 18;7(37):59429–40. doi: 10.18632/oncotarget.10683 (PMC5312322; doi:10.18632/oncotarget.10683)
Supplement: Supplementary file 1 [file oncotarget-07-59429-s001.pdf]

IL-1β-stimulated β-catenin up-regulation promotes angiogenesis in human lung-derived mesenchymal stromal cells through a NF-κB-dependent microRNA-433 induction

SUPPLEMENTARY FIGURES

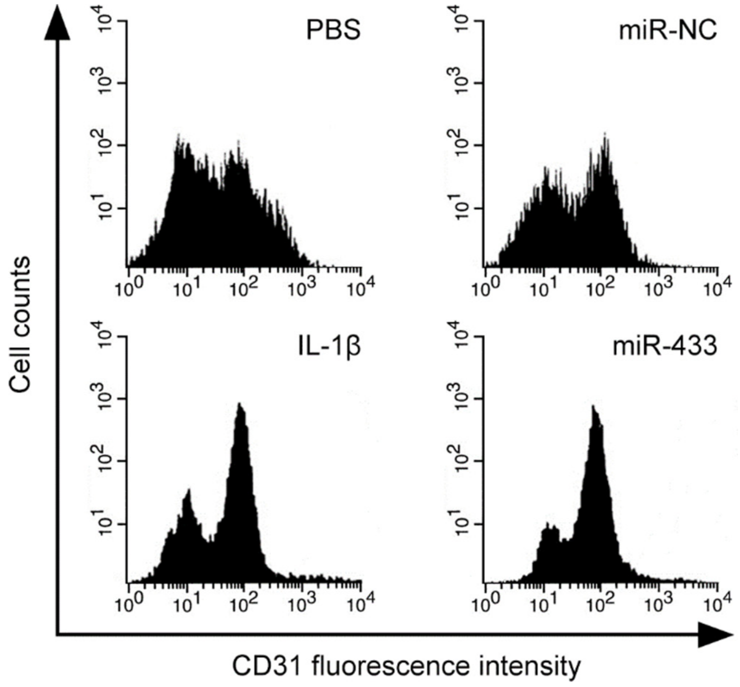

Supplementary Figure S1: Endothelial differentiation of hL-MSC was significantly enhanced by IL-1β treatment and miR-433 transfection. hL-MSC culture after IL-1β treatment (PBS as control) or miR-433 transfection (miR-NC as control), respectively, were subjected to endothelial differentiation, and then characterized by flow cytometry using FITC-conjugated CD31 antibody.

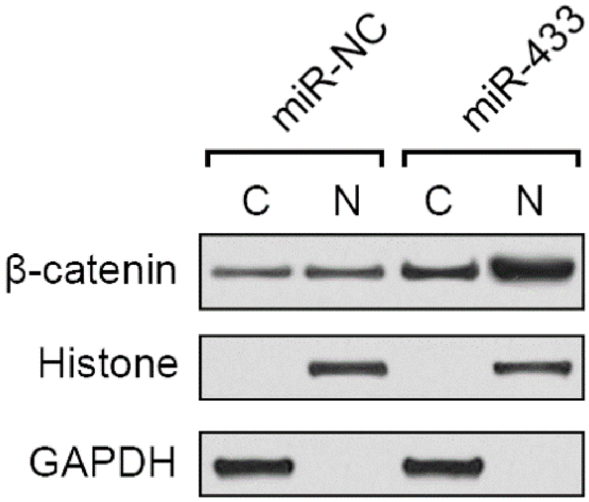

Supplementary Figure S2: Nuclear import of β-catenin was upregulated by miR-433. hL-MSC were transfected with either miR-NC or miR-433, and subjected to fractionation. Protein levels of β-catenin in the cytosolic (C, indicated by GAPDH) and nuclear (N, indicated by histone H3) fractions were analyzed by Western blot.
